# Locoregional delivery of CAR T cells in high-grade gliomas: a systematic analysis of safety, efficacy, and emerging biomarkers of response

**DOI:** 10.1136/jitc-2025-014450

**Published:** 2026-03-27

**Authors:** Christian K Ramsoomair, Manav Daftari, Rami Benchouia, Dagoberto Estevez-Ordonez, Anurag Aka, Vratko Himic, Manuela Aramburu-Berckemeyer, Daniel Kreatsoulas, Vaidya Govindarajan, Jay S Chandar, Michael E Ivan, Ricardo J Komotar, Ashish Shah

**Affiliations:** 1 University of Miami Department of Neurological Surgery, Miami, Florida, USA; 2 Medical Scientist Training Program, University of Miami Leonard M Miller School of Medicine, Miami, Florida, USA; 3 Sylvester Comprehensive Cancer Center, University of Miami, Miller School of Medicine, Miami, Florida, USA

**Keywords:** Chimeric antigen receptor - CAR, T cell, Immunotherapy, Neurotoxicity

## Abstract

**Background:**

Chimeric antigen receptor T-cell (CAR-T) therapy represents a promising frontier in oncology, but its application to high-grade gliomas (HGG) is challenged by the blood-brain barrier, limited efficacy, and significant toxicities associated with systemic administration. Locoregional delivery has the potential to address these shortcomings. This systematic review evaluates the safety and efficacy of locoregional vs systemic CAR-T cell delivery for HGG.

**Methods:**

Following PRISMA (Preferred Reporting Items for Systematic Reviews and Meta-Analyses) guidelines, a total of 112 studies were identified from three separate databases between 2015 and 2024. Of these, 19 articles were assessed for eligibility, resulting in 16 articles meeting the inclusion criteria with 194 treated patients across 14 clinical trials. A comparative meta-analysis was performed to compare the safety and efficacy outcomes of locoregional administration (eg, intracerebroventricular, intratumoral) with systemic (intravenous) delivery. Severe (grade ≥3) adverse event rates and therapeutic responses were pooled to calculate crude incidence, rate ratios, and relative risks (RRs) with 95% CIs. Both fixed-effect and random-effects models were used to evaluate incidence rate ratios.

**Results:**

Locoregional delivery was associated with a markedly improved safety profile, demonstrating an over 60% reduction in the incidence of grade ≥3 adverse events compared with systemic infusion (RR=0.39; 95% CI 0.30 to 0.52; p<0.001). Furthermore, locoregional strategies demonstrated encouraging signals of antitumor activity, including rates of disease responses not widely observed with systemic approaches (RR=3.79; 95% CI 1.23 to 11.70; p<0.05). Locoregional delivery also enables the analysis of cerebrospinal fluid to monitor T-cell trafficking and emerging biomarkers of immune activation.

**Conclusion:**

Intracranial delivery of CAR-T cells helps overcome key barriers that limit the efficacy and safety of systemic therapy in brain tumors. These findings support a paradigm shift that integrates locoregional delivery techniques as a pivotal component in the design of future CAR-T cell trials, offering a safer and potentially more effective therapeutic approach with greater opportunities for longitudinal sampling for patients with HGG.

**WHAT IS ALREADY KNOWN ON THIS TOPIC**

- Systemic chimeric antigen receptor (CAR) T-cell therapy for high-grade gliomas has shown limited efficacy and carries substantial toxicity, in part because of poor penetration across the blood-brain barrier. Locoregional delivery has been increasingly used as a strategy in clinical trials to improve therapeutic access to the tumor microenvironment, but comparative clinical evidence has remained sparse.

**WHAT THIS STUDY ADDS**

- This systematic review and meta-analysis demonstrate that locoregional administration is associated with substantially fewer severe adverse events and shows early signs of improved antitumor activity compared with systemic infusion. It also highlights the unique ability of locoregional approaches to permit cerebrospinal fluid sampling for real-time monitoring of immune activation.

**HOW THIS STUDY MIGHT AFFECT RESEARCH, PRACTICE OR POLICY**

- These findings support the incorporation of neurosurgical delivery techniques into future CAR T cell trial designs for high-grade gliomas. The results may guide investigators toward prioritizing locoregional strategies to improve safety, enhance biological insight, and potentially increase therapeutic benefit.

## Background

 Chimeric antigen receptor T-cell (CAR-T) therapy has revolutionized the treatment of hematologic malignancies, demonstrating remarkable success in relapsed and refractory cases.^1^ However, its translation to solid tumors, particularly central nervous system (CNS) malignancies, such as glioblastoma (GBM) and diffuse midline glioma (DMG), has faced considerable challenges.^2^ Systemic administration of CAR-T cells has been hindered by several barriers, including the blood-brain barrier (BBB), poor solid tumor infiltration, and systemic toxicities such as cytokine release syndrome (CRS).^3^ These limitations necessitate the exploration of alternative delivery methods to enhance efficacy and safety.

Locoregional delivery of CAR-T cells through a neurosurgical procedure, via intracavitary/tumorous (ICT) or intracerebroventricular (ICV), presents a promising strategy to overcome the shortcomings of systemic (intravenous) infusion. Whereas ICT delivers treatment directly to the tumor site, ICV delivery introduces cells into the cerebrospinal fluid (CSF) via the ventricles to achieve broader CNS distribution. Preclinical and early clinical studies suggest that localized administration enhances CAR-T cell persistence, increases tumor infiltration, and minimizes systemic toxicities.^4 5^ By directly delivering CAR-T cells into the tumor cavity or the CSF, these approaches circumvent the BBB and optimize interactions with the tumor microenvironment.

One of the primary challenges for CAR-T therapy in gliomas is the lack of frequent and specific surface target antigens. Both adult and pediatric gliomas are notorious for their cellular and molecular heterogeneity, which lends itself to tumor recurrence via partial or complete loss of antigen expression. In the context of CAR-T therapy, this phenomenon is known as tumor antigen escape and is well-characterized in numerous hematological malignancies.^6^ However, it has most recently been shown in GBM after treatment with CAR targets such as IL-7Rα or EGFRvIII.^7 8^ Thus, current CAR-T cell therapy clinical trials for brain tumors continue to explore the targeting of a wide array of tumor antigens, including B7-H3, EGFRvIII, CD70, chlorotoxin, HER2, IL-13Rα2, IL-7Rα, GD2, MMP-2, and NKGD.

This systematic review aims to critically assess and compare the safety and efficacy of locoregionally administered CAR-T therapies with traditional systemic delivery for brain tumors. By analyzing published clinical trial reports, we seek to demonstrate the benefits of integrating neurosurgical approaches into future CNS-related CAR-T and combined immunotherapy trials.

## Methods

### Search strategy and eligibility criteria

Following PRISMA (Preferred Reporting Items for Systematic Reviews and Meta-Analyses) guidelines, a comprehensive literature search was conducted using PubMed, Embase, and Scopus to identify studies using CAR-T cells to treat CNS tumors comparing systemic and locoregional delivery (online supplemental Figure 1). In addition to database searches, reference lists of included articles and ClinicalTrials.gov were manually screened to identify additional eligible studies. The following search strategy was used and adapted for each database: (“Glioblastoma”[MeSH] OR glioblastoma OR GBM OR “high-grade glioma” OR “diffuse midline glioma” OR DMG OR DIPG OR “diffuse intrinsic pontine glioma” OR “brain tumor” OR “brain cancer”) AND (“Chimeric Antigen Receptor T-Cells”[MeSH] OR “CAR T” OR “chimeric antigen receptor T” OR “CAR T-cell therapy” OR “T cell immunotherapy”) AND ((“systemic” OR “intravenous” OR IV) OR (“loco-regional” OR locoregional OR intratumoral OR “intra-tumoral” OR intracerebroventricular OR ICV OR “regional administration”)). Filters were applied to ensure inclusion of original research, full-length articles, and studies published in English. The search was performed with no restrictions on publication date. Inclusion criteria require that each study included clinical trials for CNS neoplasms, involved administration of CAR-T cell therapy, and reported clinical outcomes. Studies were excluded if they were not original clinical research, did not involve CNS tumors, did not evaluate CAR-T therapy, or lacked outcome data relevant to this review. Two reviewers independently screened titles and abstracts to assess eligibility. Any disagreements were resolved through consensus decision.

#### Data extraction

Two reviews independently extracted data from eligible studies. Extracted data included study design and phase, CAR-T construct and target antigen, route of administration, patient demographics, and reported outcomes. Primary outcomes of this study include adverse effects associated with CAR-T therapy reported as incidence (IE). In this study, IE is defined as the number of events per patient. Secondary outcomes included efficacy, as measured by overall survival (OS) and progression-free survival (PFS).

For analytical consistency, studies were categorized according to the route of CAR-T cell administration. The systemic cohort included studies in which CAR-T cells were delivered intravenously. The locoregional cohort included studies that used direct intracranial administration, such as intratumoral, intracavitary, or intraventricular delivery. Studies employing combined or sequential delivery, defined as both systemic and locoregional infusions within the same protocol, were grouped with the locoregional cohort due to the small number of such studies (n=2), which precluded separate analysis. Patients who did not receive locoregional infusions were excluded, and adverse events were attributed to systemic or locoregional delivery accordingly.

### Statistical analysis

Data from included studies were tabulated to compare baseline characteristics, delivery methods, and outcomes between systemic and locoregional CAR-T administration. Adverse events (AEs) were summarized as counts and proportions. Severe (grade ≥3) AE rates and responses to therapy were aggregated across studies within each delivery route to calculate pooled crude incidence, corresponding rate ratios and relative risk with 95% CIs. For all comparative analyses, the systemic delivery cohort was designated as the control group. Survival outcomes were extracted as reported in the original studies such as median OS or PFS, HRs, or Kaplan-Meier estimates. All statistical analyses were conducted using R (V.4.4.1) with the packages *metafor, dplyr, readxl, and writexl*. Statistical significance was defined as p<0.05.

Both fixed‐effect and random‐effects models were employed to evaluate the incidence rate ratios of clinical outcomes across delivery routes. The fixed‐effect model assumed a common true effect size and provided crude relative risks (RRs) calculated from pooled event counts, while the random‐effects model incorporated a generalized linear mixed modeling framework to account for between‐study variability.

## Results

### Study characteristics

**Figure 1:**
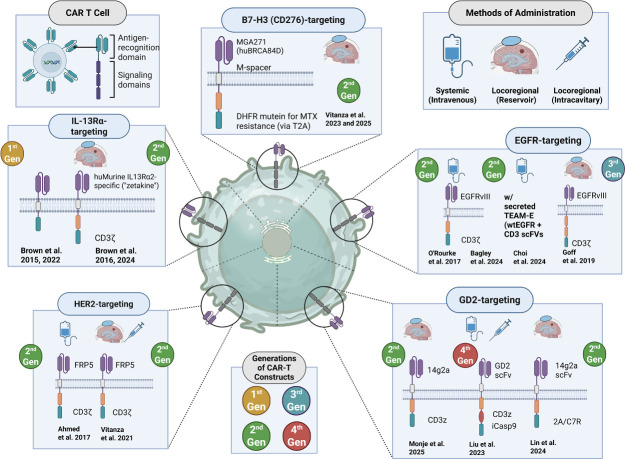
Target antigens, CAR-T construct designs, and delivery methods in glioma clinical trials. Schematic overview of CAR-T constructs evaluated in glioma, highlighting targeted antigens, generational design, and routes of administration. Top left: structural components of CAR-T cells, including antigen-recognition and signaling domains. Top right: delivery strategies used in clinical trials, including systemic (intravenous), locoregional reservoir-based (eg, intraventricular via Ommaya), and locoregional intracavitary administration. Panels: representative constructs and trial designs targeting IL-13Rα2 (Brown *et al* 2015^22^, 2016^7^, 2022^23^), 2024 HER2 (Ahmed *et al.*^11^; Vitanza *et al.* 2021),^19^ B7-H3 (CD276) (Vitanza *et al.* 2023^25^, 2025^13^) EGFR/EGFRvIII (O’Rourke *et al.* 2017, Bagley *et al.* 2024^10^, Choi *et al.* 2024^24^, Goff *et al.* 2019^9^) and GD2 (Monje *et al.* 2025;^16^ Liu *et al.* 2023;^26^ Lin *et al.* 2021).^20^ Constructs range from first-generation CAR-Ts incorporating only CD3ζ signaling to second-generation designs adding co-stimulatory domains (eg, CD28, 4–1BB), third-generation constructs with multiple co-stimulatory elements, and fourth-generation “armored” CAR-Ts integrating safety switches or immune-modulatory functions (eg, iCasp9, cytokine secretion). The common central domains of CD8α, CD28, and 4–1BB have not been labeled; instead, the unique intracellular and extracellular components of the CAR-T constructs have been highlighted. CAR, chimeric antigen receptor.

A total of 16 papers from 14 registered clinical trials on CAR T-cell administration for brain tumors were included in this systematic review. These trials were all phase I studies and encompassed both adult and pediatric populations. Patient cohorts included adults primarily with GBM (most often recurrent, but some studies investigated de novo and progressive GBM), pediatric patients with diffuse intrinsic pontine glioma (DIPG) or DMG, and patients with other high-grade gliomas such as anaplastic astrocytoma and ependymoma (table 1, figure 1).

**Table 1:** CAR-T clinical trials against high-grade gliomas

NCT number	First author	Target antigen	Disease	Dose frequency	Co-therapies	Treated (n=)	CAR generation	≥grade 3 AEs
Systemic delivery								
NCT01454596	Goff et al^9^	EGFRvIII	rGBM	Single infusion	Lymphodepletion	18	Third Gen	86
NCT02209376	O’Rourke *et al*^8^	EGFRvIII	rGBM	Single infusion	No lymphodepleting chemo	10	Second Gen	9
NCT03726515	Bagley *et al*^10^	EGFRvIII	De novo GBM	Multiple infusions	No lymphodepleting chemo+pembrolizumab	7	Second Gen	11
NCT01109095	Ahmed *et al*^11^	HER2	Progressive GBM	Multiple infusions	No lymphodepleting chemo	17	Second Gen	10
NCT04099797	Lin *et al*^20^	GD2	DMG/rCNS tumors	Multiple infusions	Lymphodepletion	11	Second Gen	1
Locoregional delivery								
NCT00730613	Brown *et al*^22^	IL-13Rα2	rHGG/ rGBM	Multiple infusions	No lymphodepleting chemo	3	First Gen	6
NCT01082926 (2022)	Brown *et al*^23^	IL-13Rα2	rGBM	Multiple infusions	No lymphodepleting chemo+aldesleukin	6	First Gen	21
NCT02208362 (2016)	Brown *et al*^7^	IL-13Rα2	Multifocal rGBM with LM mets	Multiple infusions	No lymphodepleting chemo	1	Second Gen	0
NCT02208362 (2024)	Brown *et al*^12^	IL-13Rα2	rHGG (majority rGBM, IDH-wt)	Multiple infusions	No lymphodepleting chemo	65	Second Gen	23
NCT03500991	Vitanza *et al*^19^	HER2	r/r CNS tumors	Multiple infusions	No lymphodepleting chemo	3	Second Gen	4
NCT05660369	Choi *et al*^24^	EGFRvIII+EGFRwt	rGBM	Single infusion	No lymphodepleting chemo+anakinra	3	Second Gen+TEAMs	2
NCT05168423	Bagley *et al*^10^	EGFR+IL-13Rα2	rGBM (IDH-wt)	Single infusion	No lymphodepleting chemo+anakinra	6	Second Gen (bicistronic)	11
NCT04185038 (2023)	Vitanza *et al*^25^	B7-H3 (CD276)	DIPG	Multiple infusions	No lymphodepleting chemo	4	Second Gen	0
NCT04185038 (2025)	Vitanza *et al*^13^	B7-H3 (CD276)	DIPG	Multiple infusions	No lymphodepleting chemo	21	Second Gen	11
Combined/sequential delivery								
NCT03170141	Liu *et al*^26^	GD2	GBM	Multiple infusions	Lymphodepletion	8	Fourth Gen (iCasp9)	2
NCT04196413	Monje *et al*^16^	GD2	DIPG/sDMG	Multiple infusions	Lymphodepletion	11	Second Gen	21

AEs, adverse events; CAR, chimeric antigen receptor; CNS, central nervous system; DIPG, diffuse intrinsic pontine glioma; DMG, diffuse midline glioma; GBM, glioblastoma; Gen, generation; IDH-wt, isocitrate dehydrogenase wild-type; LM, leptomeningeal ; rCNS, recurrent central nervous system tumors; rHGG, recurrent high-grade glioma; r/r, relapsed/refractory; sDMG, spinal diffuse midline glioma; TEAMs, T-cell–engaging antibody molecules.

**Figure 2:**
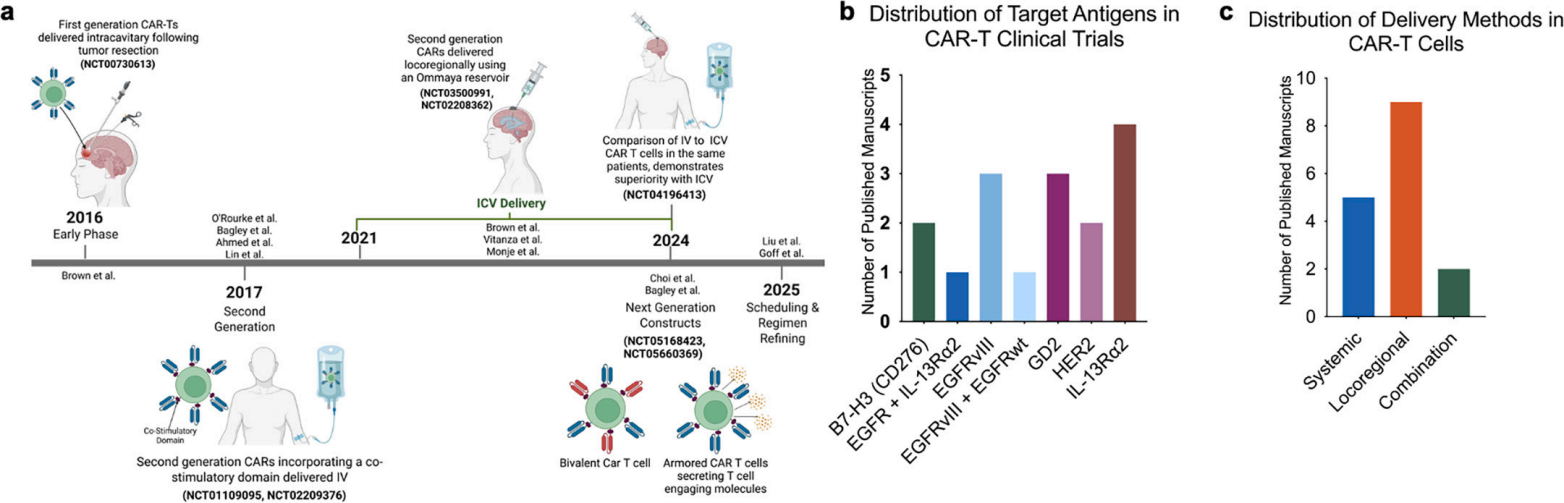
CAR-T clinical trials for glioma: evolution, antigens, and delivery strategies. (a) Timeline summarizing the development of CAR-T therapy for glioma, beginning with first-generation constructs delivered intracavitary after resection (2016), followed by second-generation CARs incorporating co-stimulatory domains administered intravenously (2017), and more recent locoregional approaches using intraventricular or intratumoral delivery via Ommaya reservoirs (2021–2024). Next-generation constructs, including bivalent and armored CAR-T cells, are currently entering early-phase studies (2025). Representative clinical trial identifiers are indicated. (b) Distribution of target antigens tested in glioma CAR-T published clinical manuscripts, including B7-H3, EGFR/EGFRvIII, HER2, GD2, and IL-13Rα2, as well as multi-antigen and combinatorial strategies. (c) Delivery methods employed across glioma CAR-T published clinical manuscripts, demonstrating relative frequencies of systemic, locoregional, and combined administration routes. CAR, chimeric antigen receptor; ICV, intracerebroventricular.

Therapeutic strategies to enhance CAR-T treatment against CNS tumors encompass both pharmacologic approaches and innovative delivery methods aimed at improving antitumor efficacy (figure 2a). Advances in CAR T-cell design have focused on overcoming resistance mechanisms and augmenting T-cell function within the tumor microenvironment. Seven distinct targets or antigen combinations were identified across published manuscripts. The most frequently targeted antigen was IL-13Rα2 (n=4), followed by EGFRvIII (n=3) and GD2 (n=3). Other targets included HER2 (n=2), B7-H3 (n=2), and bivalent constructs targeting EGFR+IL-13Rα2 (n=1) and EGFRvIII+EGFRwt (n=1) (figure 2b).

Regarding the route of administration, nine published manuscripts (64.3%) employed an exclusively locoregional delivery approach, five used only systemic (intravenous) delivery, and two explored combined or sequential strategies (figure 2c). Locoregional approaches included ICT and/or ICV. Among the nine locoregional trials included, five employed intracavitary (ICT) delivery, three used ICV delivery, and one incorporated a combination of both approaches. Use of an Ommaya reservoir was the most common method of locoregional delivery (n=5), followed by Rickham reservoir (n=4). Although these delivery methods share similar objectives, each has distinct advantages and limitations depending on tumor location and disease context. All trials involving brainstem and spinal gliomas used ICV delivery, reflecting the relative proximity of these tumors to CSF pathways and the increased risk associated with repeated direct intratumoral injections in these regions. In contrast, all trials involving GBM employed ICT delivery. GBMs, which are typically characterized by aggressive, bulky disease typically located away from CSF spaces, are often well suited for intracavitary administration. Notably, one trial involving patients with multifocal leptomeningeal GBM employed a combined ICT and ICV delivery strategy to address both parenchymal and leptomeningeal disease components. Delineating any heterogeneity between trial methods, an overview of CAR-T cell dosing ranges, number of infusions, and administration intervals is presented in online supplemental Table 1.

The use of lymphodepletion and other co-therapies was highly dependent on the route of administration. For trials investigating systemic delivery, three studies (60%) incorporated co-therapies, including lymphodepleting chemotherapy (eg, cyclophosphamide and fludarabine), supplemental interleukin (IL)-2 infusions, or immune checkpoint inhibitors like pembrolizumab. Similarly, two trials (100%) that evaluated a combined systemic and locoregional delivery paradigm employed lymphodepletion. In contrast, trials using an exclusively locoregional delivery strategy did not report use of lymphodepletion or other supportive agents, suggesting that direct CNS delivery may obviate the need for systemic immune conditioning and its associated toxicities.

### Clinical outcomes

**Figure 3:**
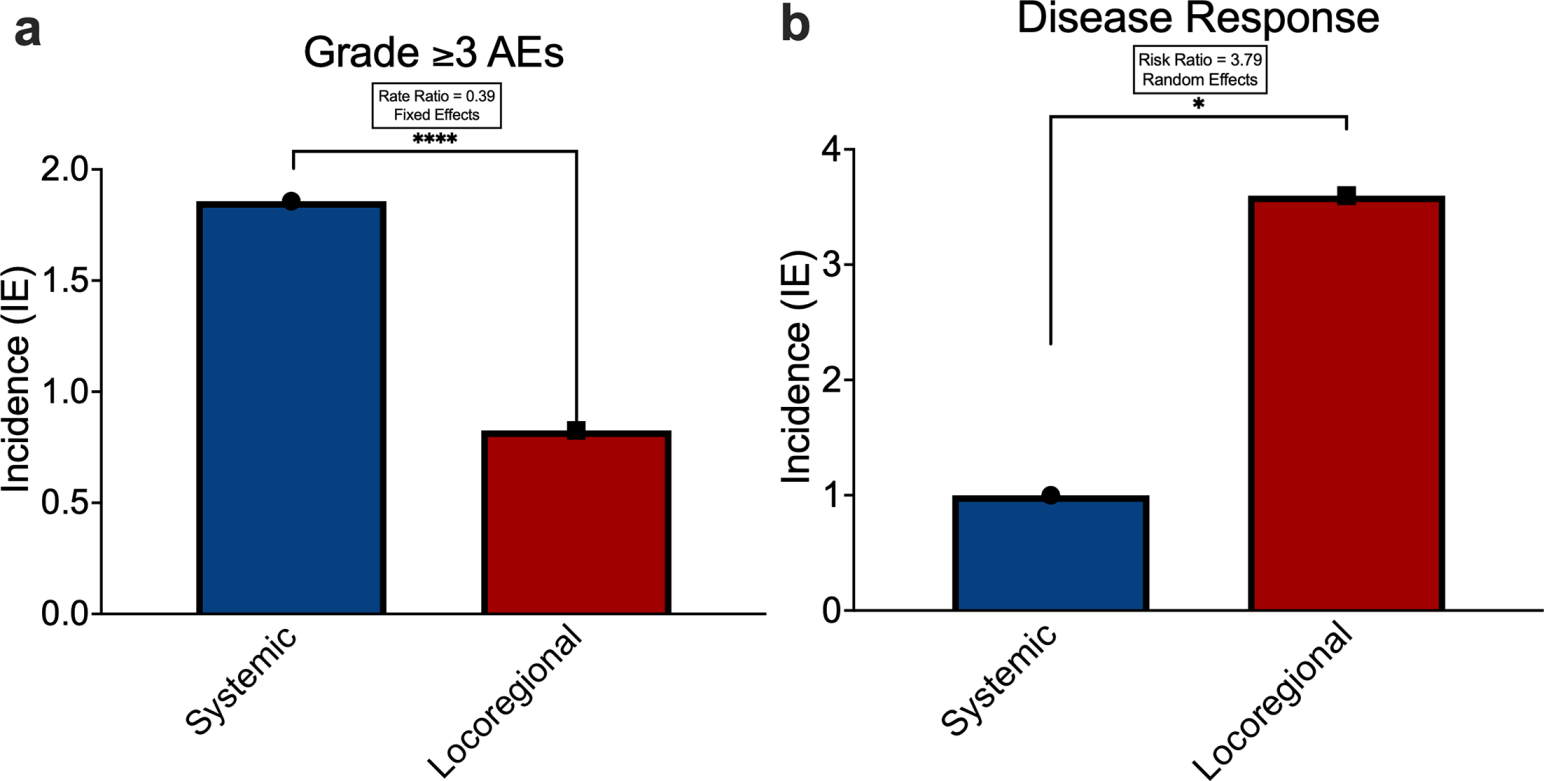
Incidence of severe AEs and stable disease in CAR-T cell clinical trials for glioma. (a) Incidence of grade ≥3 AEs by delivery modality, comparing systemic and locoregional approaches. Fixed effects model stated RR=0.39; 95% CI 0.30 to 0.52; p<0.001. (b) Incidence rate of disease response (CR, PR, and SD per RANO guidelines) by delivery modality. A random effects model, which accounts for trial heterogeneity, stated RR=3.79; 95% CI 1.23 to 11.70; p<0.05. AEs, adverse events; CAR, chimeric antigen receptor; CR, complete response; PR, partial response; RANO, Response Assessment in Neuro-Oncology; RR, relative risk; SD, stable disease.

Next, we analyzed the rate of grade ≥3 AEs by delivery method. The highest burden of severe AEs was observed in the systemic cohort, which reported 117 events among 63 patients (1.86 AEs per patient) across five studies, with a median AE follow-up duration of 1.0 month (IQR 1.0–1.0). The locoregional cohort reported 78 events among 107 patients (IE=0.729 AEs per patient) across seven trials, with a median AE follow-up duration of 1.0 month (IQR 1.0–2.0). Grade ≥3 AE rates were then aggregated across studies and compared by delivery route. Locoregional delivery was associated with an over 60% lower incidence of grade ≥3 AEs compared with systemic administration (RR=0.39; 95% CI 0.30 to 0.52; p<0.001)**,** representing a statistically significant reduction in a fixed‐effect model (figure 3a). In contrast, the random-effects model did not yield statistically significant results, likely due to limited power when accounting for intertrial heterogeneity. To further evaluate potential bias inherent to the fixed-effect assumption, we performed an additional analysis comparing AEs across CAR antigen targets irrespective of delivery method. No CAR antigen target was associated with a significantly lower AE rate in the fixed-effect model (online supplemental Materials). Taken together, these analyses support the fixed-effect estimate as a reasonable assessment for exploratory reporting. Nevertheless, these findings should be interpreted cautiously and considered hypothesis-generating pending validation in larger cohorts and additional studies.

Given the favorable safety profile observed with locoregional delivery, we further analyzed the clinical efficacy regarding this approach. Response to therapy was assessed via the Response Assessment in Neuro-Oncology (RANO) criteria and categorized as complete response (CR), partial response (PR), or stable disease (SD). All studies included in this analysis were verified to adhere to RANO response criteria, with only five locoregional studies excluded on this basis. Under RANO, CR is defined as disappearance of all enhancing disease with stable or improved non-enhancing disease, no new lesions, stable or improving clinical status, and no corticosteroid requirement. PR is defined as a ≥50% reduction in enhancing tumor burden with no progression of non-enhancing disease, stable or reduced corticosteroid use, and stable or improved clinical status. SD is defined as not meeting criteria for CR, PR, or progressive disease, in the setting of stable imaging, neurologic status, and corticosteroid use.

Incidence of CR was highest in the locoregional delivery cohort, with three CRs among 86 treated patients (IE=0.035). No CRs were reported in the systemic delivery cohort (IE=0 of 63). PR was also most frequent in the locoregional cohort, with eight PRs among 86 patients (IE=0.093), followed by the systemic cohort, with three PRs among 63 patients (IE=0.048). The incidence of SD was again highest in the locoregional delivery cohort, with 34 cases among 86 patients (IE=0.395), compared with the systemic delivery cohort, which reported 13 cases among 63 patients (IE=0.206). To assess disease response across locoregional and systemic cohorts, response rates were pooled by delivery route using a random-effects model, appropriately accounting for inter-trial heterogeneity. Locoregional delivery was associated with a significantly higher incidence of disease response (CR+PR+SD) relative to systemic administration (RR=3.79; 95% CI 1.23 to 11.70; p<0.05) (figure 3b). All raw model outputs and supporting analyses are provided in online supplemental Materials. These findings collectively demonstrate a trend toward improved disease control with locoregional administration.

Further prognostic analyses were limited by incomplete reporting of neurologic symptom changes, median overall survival (mOS), and median progression-free survival (mPFS), particularly in locoregional trials. No locoregional studies reported mOS, and only one locoregional trial reported mPFS.

## Discussion/conclusion

### Locoregional delivery appears to mitigate systemic toxicity

High levels of toxicities associated with systemic delivery of CAR-Ts have been reported in several CNS tumor studies. For example, systemic EGFRvIII-targeted trials documented 86 grade ≥3 AEs, including one treatment-related death, from a single study.^9^ Other trials reported severe neurologic events like cerebral edema and facial muscle weakness.^8 10^ Similarly, an HER2-targeted intravenous trial reported grade 4 toxicities, including cerebral edema.^11^

In contrast, the majority of recent locoregional CAR-T trials have shown impressive safety profiles. The largest brain tumor CAR-T trial to date (NCT02208362, Brown *et al*) reported no dose-limiting toxicities (DLTs) across 58 patients receiving IL-13Rα2 CAR-T cells.^12^ Similarly, a pediatric B7-H3 trial (NCT04185038) demonstrated the tolerability of repetitive, high-dose ICV infusions with only a single DLT reported.^13^

As the CAR-T field in neuro-oncology has matured, neurotoxicities have become increasingly recognized and stratified. Two major categories have emerged as central for optimizing safety and advancing translation and include immune effector cell-associated neurotoxicity syndrome (ICANS) and tumor inflammation-associated neurotoxicity (TIAN).^14^ Often preceded by CRS, ICANS is a global cytokine-mediated neurotoxicity syndrome, usually developing 1 week after infusion.^15^ In contrast, TIAN reflects localized, secondary on-target inflammation at the tumor site. Importantly, the incidence and severity of these toxicities are shaped not only by immune activation, but also by delivery strategy and associated treatment variables.

**Figure 4:**
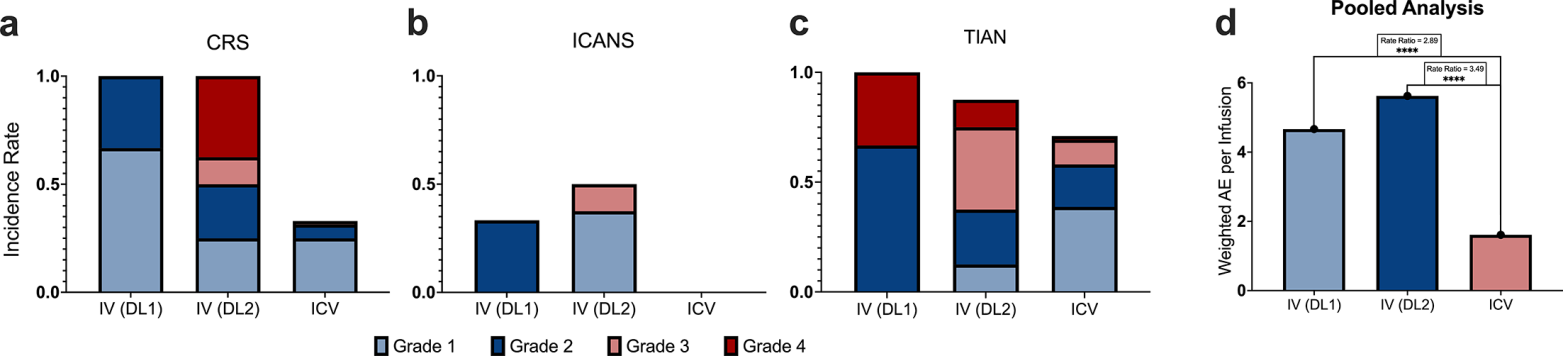
Safety comparison of GD2 CARs by delivery route in matched patients. Comparison of adverse event per infusion between IV (DL1, 1×10⁶ kg⁻¹; DL2, 3×10⁶ kg⁻¹) and ICV (10–30×10⁶) CAR T-cell delivery in trial NCT04196413 of CRS, (a), ICANS, (b), and TIAN, (c) are shown separately to illustrate toxicity patterns. Statistical significance reflects pooled and weighted (by grade) adverse events across all toxicity categories (d). CAR, chimeric antigen receptor; CRS, cytokine release syndrome; DL, dose level; ICANS, immune effector cell-associated neurotoxicity syndrome; ICV, intracerebroventricular; IV, intravenous; TIAN, tumor inflammation-associated neurotoxicity.

Notably, none of the locoregional trials included here employed conditioning chemotherapy, whereas 47% of patients in the systemic cohort did. This discrepancy has prompted debate as to whether the improved safety profiles observed with locoregional delivery may be attributable to the absence of lymphodepletion alone. However, evidence from trials incorporating both systemic and locoregional administration suggests that delivery route itself plays an important role. In 2024, the GD2-targeted CAR T-cell trial by Monje *et al* (NCT04196413) provided a direct intrapatient comparison between intravenous and ICV delivery.^16^ Initial intravenous administration led to dose-limiting CRS and numerous high-grade AEs of ICANS and TIAN.^16 17^ By contrast, subsequent ICV infusions in the same patients were exceptionally well-tolerated, with no DLTs, no ICANS events, and only mild, manageable CRS reported.^16^ Using ICV delivery as the reference, we performed a grade-weighted pooled analysis that demonstrated significantly higher CRS, ICANS, and TIAN toxicity with intravenous delivery at both dose levels compared with ICV administration (intravenous dose level 1 (DL1): RR 2.89, 95% CI 1.65 to 5.06; intravenous DL2: RR 3.49, 95% CI 2.45 to 4.96; p<0.001) (figure 4).

This improved safety profile is not merely an observation but is grounded in key pharmacological principles of direct CNS delivery. Confining therapeutics to the CNS limits exposure to normal peripheral tissues that may express the target antigen, thereby lowering the risk of on-target, off-tumor toxicity while ensuring CAR T cells are not dampened by peripheral immune regulation before reaching their target.^16 18^

### Neurosurgical delivery has the potential to unlock antitumor efficacy

Beyond improving the safety profile, locoregional delivery has demonstrated encouraging signals of antitumor activity that have been largely absent in systemic trials for CNS tumors. To date, no complete responses have been observed in any systemic-administration CAR T-cell trial for CNS tumors. In contrast, neurosurgical delivery has produced, in some cases, dramatic clinical responses. For example, Monje *et al* observed impressive tumor regression in multiple patients, including one CR and three PRs, with DIPG/DMG following locoregional ICV delivery (after intravenous infusions).^16^ In the large anti-IL-13Rα2 trial by Brown *et al*, locoregional delivery resulted in two CRs, two PRs, and stable disease or better in 50% of patients.^12^

From a neurosurgical perspective, we anticipate several ways the field will continue to evolve. First, we believe that repeated locoregional infusions will become standard to CAR T-cell therapy in neuro-oncology. While Ommaya reservoirs have enabled relatively straightforward repeated ICV administration, there remains a clear need for improved delivery platforms to safely and reliably support repeated intracavitary infusions. Second, although ICV delivery is currently favored for brainstem gliomas and leptomeningeal disease, and intracavitary delivery is more commonly used for GBM, further work is needed to rigorously define the relative efficacy and safety profiles of these approaches across disease contexts. Finally, we strongly encourage the development of technologies that enable real-time visualization and tracking of CAR T cells within the CNS. Such tools would provide critical insight into cell distribution, persistence, and on-target activity, and could meaningfully inform both surgical strategy and therapeutic optimization.

### Emerging biomarkers of response and resistance

Locoregional delivery provides the opportunity to study the CNS tumor microenvironment in real-time. Indwelling catheters like an Ommaya reservoir may serve not only as a conduit for therapy but also as a diagnostic port for the serial collection of CSF. This allows for a new class of locoregional biomarkers that link pharmacodynamics with response and resistance.

CSF cytokine profiles are powerful pharmacodynamic markers of CAR-T cell activity. In the phase I trial of IL-13Rα2 CAR-T cells (NCT02208362), administration was associated with significant increases in CSF levels of the inflammatory cytokines interferon-γ and the T-cell-attracting chemokines CXCL9 and CXCL10, confirming local immune activation.^12^ Similarly, an interim analysis of a pediatric trial of HER2-targeted CAR-T cells (NCT03500991) reported high CSF concentrations of CXCL10 and CCL2 post-infusion, providing correlative evidence of a localized immune response.^19^ In the trial of C7R-augmented GD2 CAR-T cells (NCT04099797), the development of CRS was directly correlated with increased circulating levels of IL-6 and IP-10 (CXCL10).^20^ Together, these data show that CSF cytokine analysis can serve as a liquid biopsy, providing a real-time measure of target engagement and the local inflammatory response.

Beyond soluble factors, these approaches allow for direct tracking of the therapeutic cells. CSF can be analyzed by flow cytometry to quantify the number, identity, and exhaustion status of CAR-T cells persisting over time and provide a direct measure of the therapeutic agent at its site of action. Moreover, the role of neurosurgical hardware may extend beyond locoregional delivery or sampling. In the NCT04099797 trial, CAR T cells were administered intravenously, yet Ommaya reservoirs were required, not for delivery, but as safety measures to manage therapy-induced intracranial pressure (ICP) elevations.^20^ Direct ventricular access allowed real-time ICP monitoring and CSF withdrawal for immediate relief. This underscores a key principle: as systemic therapies provoke significant CNS inflammation, integrating neurosurgical safeguards becomes essential.

Repeated CSF sampling transforms a therapeutic catheter from a delivery device into a diagnostic instrument, enabling a closed-loop “theranostic” paradigm. After each CAR T-cell infusion, CSF analysis could reveal pharmacodynamic changes such as declining T-cell counts or inflammatory markers, which would guide subsequent dosing and timing. This adaptive approach shifts high-grade glioma therapy from static protocols toward dynamic, real-time personalization driven by local biological feedback.

### Limitations

Because no standardized randomized control group was available across the included studies, the systemic cohort necessarily served as a constructed reference rather than a true comparator, introducing potential bias. Although the disease response analysis remained significant under a random-effects model, which inherently accounts for inter-trial heterogeneity, the AE analysis did not and therefore relied on fixed-effect modeling. Given that fixed-effect models do not account for between-study variability, we performed additional analyses to assess whether CAR antigen target biased these results.

In the absence of a natural reference group, IL13Rα2 was selected as the comparator, as its AE rate was closest to the overall median. Under the fixed-effect model, no CAR target was associated with a significantly lower AE rate; however, EGFRvIII-targeting and EGFRvIII+IL13Rα2-targeting CARs were associated with significantly higher AE rates (online supplemental Materials). While both systemic and locoregional trials employed EGFRvIII-targeting CARs, a greater proportion of EGFRvIII-treated patients were in the systemic cohort, whereas EGFRvIII+IL13Rα2 trials were exclusively locoregional. We therefore identify CAR target distribution as a potential confounding factor in the AE analysis.

More broadly, differences in patient populations, disease burden, prior therapies, and trial timing and design between systemic and locoregional studies may further confound direct comparisons, particularly within the constraints of a fixed-effect framework. We acknowledge that a direct comparison of systemic versus locoregional delivery using identical CAR targets and constructs would provide the most rigorous assessment of delivery-dependent effects. However, the available literature is limited by a small number of studies within individual CAR target and construct categories, which constrained our ability to perform adequately powered, target-matched subgroup analyses for treatment response.

### Future directions and conclusion

Early-phase locoregional CAR T-cell trials have yielded encouraging results, but fully realizing their potential will require harmonized trial design, standardized outcome reporting, and close interdisciplinary collaboration. Current studies in high-grade gliomas remain highly heterogeneous with respect to delivery methods, dosing schedules, response criteria, and toxicity grading, limiting cross-trial comparisons and obscuring safety signals. Establishing consensus guidelines for trial conduct and reporting will be essential to improve interpretability, reproducibility, and regulatory evaluation. Future efforts should prioritize biomarker-driven trial designs, including serial CSF sampling to track T-cell dynamics and inflammatory responses, as well as strategic intraoperative biosampling to interrogate mechanisms of response and resistance.^21^ Ultimately, the success of locoregional CAR T-cell therapy will depend on coordinated expertise spanning neurosurgery, neuro-oncology, immunology, radiology, and bioengineering. Sustained multidisciplinary collaboration will be essential to translating early promise into durable clinical benefit and to refining both cellular products and delivery strategies. We envision that continued progress in locoregional CAR T delivery will catalyze broader neurosurgical applications of cell-based and gene-modified therapies, offering hope for patients with intractable brain tumors.

## Supplementary material

10.1136/jitc-2025-014450 online supplemental file 1

10.1136/jitc-2025-014450 online supplemental file 2

## Data Availability

All data relevant to the study are included in the article or uploaded as supplementary information.
