# Supplementary material for: Locoregional delivery of CAR T cells in high-grade gliomas: a systematic analysis of safety, efficacy, and emerging biomarkers of response
Source: J Immunother Cancer. 2026 Mar 27;14(3):e014450. doi: 10.1136/jitc-2025-014450 (PMC13034252; doi:10.1136/jitc-2025-014450)
Supplement: online supplemental file 1 [file jitc-14-3-s001.pdf]

Supplemental Materials

Full Table 1 (Summary Table Included in Manuscript)

NCT Number	First Author	Target Antigen	Disease	Dose Frequency	Co-therapies	Treated (n=)	CAR Generation	≥ Grade 3 AEs	Median Follow-up (mOS)	CR	PR	SD
**Systemic Delivery**												
**NCT01454596**	Goff et al.	EGFRvIII	rGBM	single infusion	Lymphodepletion	18	3rd Gen	86	NR	0	0	0
**NCT02209376**	O'Rourke et al.	EGFRvIII	rGBM	single infusion	No lymphodepleting chemo	10	2nd Gen	9	NR	0	0	1
**NCT03726515**	Bagley et al.	EGFRvIII	de novo GBM	multiple infusions	No lymphodepleting chemo + Pembrolizumab	7	2nd Gen	11	2.5	0	0	0
**NCT01109095**	Ahmed et al.	HER2	progressive GBM	multiple infusions	No lymphodepleting chemo	17	2nd Gen	10	8	0	1	7
**NCT04099797**	Lin et al.	GD2	DMG / rCNS tumors	multiple infusions	Lymphodepletion	11	2nd Gen	1	NR	0	2	5
**Locoregional Delivery**												
**NCT00730613**	Brown et al.	IL-13Rα2	rHGG / rGBM	multiple infusions	No lymphodepleting chemo	3	1st Gen	6	NR	0	0	0
**NCT01082926**	Brown et al. (2022)	IL-13Rα2	rGBM	multiple infusions	No lymphodepleting chemo + Aldesleukin	6	1st Gen	21	NR	0	0	0
**NCT02208362 (2016)**	Brown et al. (2016)	IL-13Rα2	multifocal rGBM with LM mets	multiple infusions	No lymphodepleting chemo	1	2nd Gen	0	NR	1	0	0
**NCT02208362 (2024)**	Brown et al. (2024)	IL-13Rα2	rHGG (majority rGBM, IDH-wt)	multiple infusions	No lymphodepleting chemo	65	2nd Gen	23	NR	2	2	26
**NCT03500991**	Vitanza et al.	HER2	r/r CNS tumors	multiple infusions	No lymphodepleting chemo	3	2nd Gen	4	NR	0	0	1
**NCT05660369**	Choi et al.	EGFRvIII + EGFRwt	rGBM	single infusion	No lymphodepleting chemo + Anakinra	3	2nd Gen + TEAMs	2	NR	0	3	0
**NCT05168423**	Bagley et al.	EGFR + IL-13Rα2	rGBM (IDH-wt)	single infusion	No lymphodepleting chemo + Anakinra	6	2nd Gen (bicistronic)	11	2.5	0	0	3
**NCT04185038 (2023)**	Vitanza et al. (2023)	B7-H3 (CD276)	DIPG	multiple infusions	No lymphodepleting chemo	4	2nd Gen	0	NR	0	0	1
**NCT04185038 (2025)**	Vitanza et al. (2025)	B7-H3 (CD276)	DIPG	multiple infusions	No lymphodepleting chemo	21	2nd Gen	11	NR	0	1	15

**Combined/Sequential Delivery**												
**NCT03170141**	Liu et al.	GD2	GBM	multiple infusions	Lymphodepletion	8	4th Gen (iCasp9)	2	NR	0	4	1
**NCT04196413**	Monje et al.	GD2	DIPG / sDMG	multiple infusions	Lymphodepletion	11	2nd Gen	21	NR	1	3	4

Supplemental Figure 1

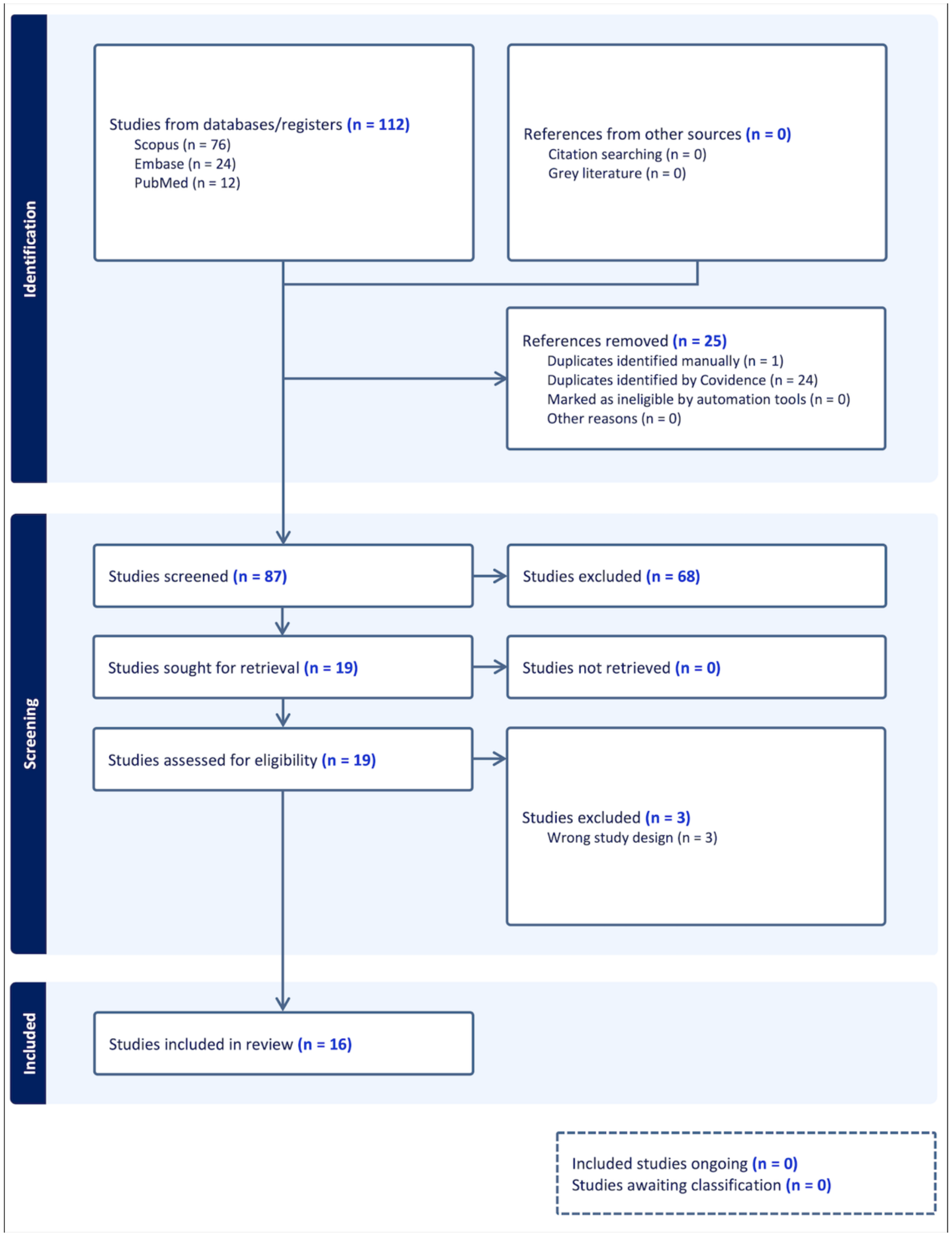

Supplemental Figure 1, PRISMA flow diagram of study selection.

A total of 112 records were identified through database searching (Scopus, n = 76; Embase, n = 24; PubMed, n = 12). After removal of 25 duplicates, 87 records were screened. Of these, 19 full-text articles were assessed for eligibility, with 3 excluded due to inappropriate study design. Ultimately, 16 studies met inclusion criteria and were incorporated into the review. No additional records were retrieved from citation searching or grey literature, and no studies were ongoing or awaiting classification.

Supplemental Table 1

NCT Number	First Author	Target Antigen	Disease	Dose (Min-Max)	Dose Frequency (#)	Co-therapies
Systemic Delivery						
NCT01454596	Goff et al.	EGFRvIII	rGBM	$1{\times}10^{7}$-$6{\times}10^{10}$/kg	single infusion	Lymphodepletion
NCT02209376	O'Rourke et al.	EGFRvIII	rGBM	$1.75{\times}10^{8}$–$5{\times}10^{8}$/kg	single infusion	No lymphodepleting chemo
NCT03726515	Bagley et al.	EGFRvIII	de novo GBM	$1{\times}10^{7}$–$2.5{\times}10^{8}$/kg	multiple infusions	No lymphodepleting chemo + Pembrolizumab
NCT01109095	Ahmed et al.	HER2	progressive GBM	$1{\times}10^{6}$–$1{\times}10^{8}$/kg	multiple infusions	No lymphodepleting chemo
NCT04099797	Lin et al.	GD2	DMG / rCNS tumors	$1{\times}10^{7}$–$3{\times}10^{7}$/kg	multiple infusions	Lymphodepletion
Locoregional Delivery						

NCT00730613	Brown et al. (2015)	IL-13Rα2	rHGG / rGBM	1×10^{8}	multiple infusions	No lymphodepleting chemo
NCT01082926	Brown et al. (2022)	IL-13Rα2	rGBM	1×10^{8}	multiple infusions	No lymphodepleting chemo + Aldesleukin
NCT02208362 (2016)	Brown et al. (2016)	IL-13Rα2	multifocal rGBM with LM mets	Initial Dose: 2.6×10^{6} Follow-up Dose: 10×10^{6}	multiple infusions	No lymphodepleting chemo
NCT02208362 (2024)	Brown et al. (2024)	IL-13Rα2	rHGG (majority rGBM, IDH-wt)	2×10^{6}–200×10^{6}	multiple infusions	No lymphodepleting chemo
NCT03500991	Vitanza et al. (2021)	HER2	r/r CNS tumors	DL1: 1×10^{7} DL2: 2.5×10^{7}	multiple infusions	No lymphodepleting chemo
NCT05660369	Choi et al.	EGFRvIII + EGFRwt	rGBM	10×10^{6}	multiple infusion	No lymphodepleting chemo + Anakinra
NCT05168423	Bagley et al.	EGFR + IL-13Rα2	rGBM (IDH-wt)	DL1: 1×10^{7} DL2: 2.5×10^{7}	single infusion	No lymphodepleting chemo + Anakinra
NCT04185038 (2023)	Vitanza et al. (2023)	B7-H3 (CD276)	DIPG	DL1: 1×10^{7} DL2: 2.5×10^{7} DL3: 5×10^{7} DL4: 10×10^{7}	multiple infusions	No lymphodepleting chemo
NCT04185038 (2025)	Vitanza et al. (2025)	B7-H3 (CD276)	DIPG	DL1: 1×10^{7} DL2: 2.5×10^{7} DL3: 5×10^{7} DL4: 10×10^{7}	multiple infusions	No lymphodepleting chemo

Combined/Sequential Delivery						
NCT03170141	Liu et al.	GD2	GBM	IV: 3×10^{7}–2.1×10^{8}/kg<br>ICV: 2.6×10^{6}–6.4×10^{6}	multiple infusions	Lymphodepletion
NCT04196413	Monje et al.	GD2	DIPG / sDMG	IV: 1×10^{6}–2×10^{6}/kg<br>ICV: 10×10^{6}–50×10^{6}	multiple infusions	Lymphodepletion

Supplemental Table 1, Summary of CAR-T Dose Levels and Administration.

Overview of dosing ranges, number of infusions, and systemic versus locoregional delivery strategies used in included clinical trials.

<u>Raw Data from Analyses</u>

Adverse Events (Greater than grade 3) incidence rate ratio by delivery method

Fixed effects

delivery_route	n_grade3plus_AEs	n_patients	rate	RR_vs_systemic	CI_low	CI_high	p_value	RR_95CI
systemic	117	63	1.857	1	0.774	1.292	1	1 [0.77–1.29]
locoregional	78	107	0.729	0.393	0.295	0.523	<0.001	0.39 [0.3–0.52]

Random Effects

delivery_route	RR_vs_systemic	CI_low	CI_high	p_value	tau2	I2	RR_95CI
systemic	1				0.969658	90.20729	1.00 [NA–NA]
locoregional	0.879764	0.273457	2.830365	0.829867	0.969658	90.20729	0.88 [0.27–2.83]

Adverse Events (Greater than grade 3) incidence rate ratio via CAR type (IL13Ra2 reference)

Fixed Effects

construct_type	n_grade3plus_AEs	n_patients	rate	RR_vs_IL13Ra2	CI_low	CI_high	p_value	RR_95CI
IL13Ra2	50	74	0.676	1	0.676	1.48	1	1 [0.68–1.48]
EGFRvIII	106	35	3.029	4.482	3.202	6.274	0	4.48 [3.2–6.27]
HER2	14	20	0.7	1.036	0.573	1.874	0.906887	1.04 [0.57–1.87]
GD2	24	30	0.8	1.184	0.728	1.926	0.496414	1.18 [0.73–1.93]
B7-H3	0	4	0	0	0			0 [0–NaN]
EGFRvIII+EGFRwt	2	3	0.667	0.987	0.24	4.055	0.985149	0.99 [0.24–4.06]
EGFR+IL13Ra2	11	6	1.833	2.713	1.413	5.212	0.002724	2.71 [1.41–5.21]

Random Effects

construct_type	RR_vs_IL13Ra2	CI_low	CI_high	p_value	tau2	I2	RR_95CI
IL13Ra2	1				0.626822	87.08954	1.00 [NA–NA]
EGFRvIII	1.507188	0.392992	5.780308	0.54972	0.626822	87.08954	1.51 [0.39–5.78]
HER2	0.624622	0.129271	3.018102	0.558172	0.626822	87.08954	0.62 [0.13–3.02]
GD2	0.360375	0.084363	1.539429	0.168308	0.626822	87.08954	0.36 [0.08–1.54]
B7-H3	9.91E-09	0	Inf	0.99282	0.626822	87.08954	0 [0–Inf]
EGFRvIII+EGFRwt	0.456955	0.044803	4.660608	0.508623	0.626822	87.08954	0.46 [0.04–4.66]
EGFR+IL13Ra2	1.385919	0.2032	9.452616	0.739002	0.626822	87.08954	1.39 [0.2–9.45]

Disease Response (CR+PR+SD) by delivery method

Fixed Effects

delivery_route	n_events	n_patients	rate	RR_vs_systemic	CI_low	CI_high	p_value
systemic_ref	16	63	0.254	1	0.500091	1.999635	1
locoregional	49	86	0.57	2.243459	1.275912	3.944716	0.005013

Random Effects

delivery_route	RR_vs_systemic	CI_low	CI_high	p_value	tau2	I2	RR_95CI
systemic_ref	1				0.397191	65.77793	1.00 [NA–NA]
locoregional	3.790542	1.228214	11.69846	0.020476	0.397191	65.77793	3.79 [1.23–11.7]

Monje et al. Analyses (IV DL1 and DL2 vs ICV) (ICV reference)

delivery_method	total_aes	infusions	AE_per_infusion	IRR_vs_ICV	CI_low	CI_high	p_value	RR_95CI
IV DL1	14	3	4.666667	2.893333	1.653856	5.061732	<0.001	2.89 [1.65–5.06]
IV DL2	45	8	5.625	3.4875	2.453105	4.958067	<0.001	3.49 [2.45–4.96]
ICV	100	62	1.612903	1	0.757914	1.319412	1	1.00 [Ref]

*Note: total AEs were calculated using a weighted system based on AE grade (g1= 1, g2=2, g3=3, g4 =4)
